# Fractal analysis in different regions of a fracture surface in a dental glass-ceramic^☆^

**DOI:** 10.1016/j.dental.2025.06.008

**Published:** 2025-06-13

**Authors:** Kartikeya S. Jodha, Susana M. Salazar Marocho, John J. Mecholsky, Seth T. Lirette, Yuanyuan Duan, Jason A. Griggs

**Affiliations:** a Department of Biomedical Materials Science, University of Mississippi Medical Center, MS, USA; b Department of Materials Science and Engineering, Herbert Wertheim College of Engineering, University of Florida, USA; c Department of Data Science, University of Mississippi Medical Center, MS, USA

**Keywords:** Fractal geometry, Atomic force microscopy, Fractography, Failure analysis

## Abstract

**Objectives:**

Previous studies have reported the fractal dimension of dental ceramic fracture surfaces from mist and hackle regions. The aim of this study was to determine and compare the fractal dimensional increment between the mirror, mist, and hackle regions of lithium disilicate fracture surfaces.

**Methods:**

Nine bar-shaped specimens were prepared from lithium disilicate glass-ceramic. One face of each specimen was indented using a Knoop diamond at 10 N followed by loading in 4-point flexure until failure at a loading rate of 12.6 MPa/s to avoid environmental slow crack growth. Fracture surfaces were replicated in epoxy, and an atomic force microscope (AFM) was used to scan the replicas. Noise in scans was reduced by Laplace transform filter. The FRACTALS software was used to determine the fractal dimensional increment (D*) by the Minkowski cover algorithm.

**Results:**

Median D values (25 %, 75 % quartiles) from mirror, mist, and hackle regions were 2.14 (2.12, 2.14), 2.14 (2.12, 2.15), and 2.14 (2.12, 2.15), respectively. A multilevel mixed model with clustering on repeated measures showed that the fractal dimension between the mirror-mist (p = 0.51), mist-hackle (p = 0.90), and mirror-hackle (p = 0.43) regions are not significantly different.

**Significance:**

Fractal dimension in mirror, mist, and hackle regions of the fracture surface were not significantly different in lithia disilicate glass-ceramics. Any portion of the primary fracture surface can be analyzed using fractal analysis to investigate the conditions present at the time of failure.

## 1. Introduction

All-ceramic restorations have gained popularity in the last decade due to the growing demand for better esthetics and allergenic concerns with metal-ceramic restorations. These non-metallic materials are biocompatible, have excellent fracture and wear resistance, and possess optical properties similar to that of a natural tooth [1–9]. While porcelain-fused-to-metal (PFM) crowns have a steady market share [10] due to their longevity, the utilization of all-ceramic restorations is increasing at a markedly higher rate. The U.S. market share for all-ceramic restorations was valued at $385.6 million in 2024 and is projected to grow at a compound annual growth rate of 5.6 % from 2024 to 2034 [11]. Advancements in digital dentistry using intraoral scanners to create virtual impressions and allow real-time adjustments, followed by more rapid fabrication of restorations using CAD/CAM systems, have further pushed the market towards ceramic-based materials [11,12].

Ceramics are brittle and susceptible to fracture as compared to their metal counterparts. Studies have reported a five-fold increase in the incidence of cusp chipping and about four times the incidence of major fracture in five years with all-ceramic fixed dental prostheses (FDP) compared to traditional metal-ceramic FDPs [13]. Systematic reviews on zirconia-based crowns and FDP have reported a success rate of 69.8 % after 3 years [14] and just 52.7 % after 9 years of use [15]. Garling et al. analyzed FDPs fabricated from lithium disilicate glass-ceramic and observed a decline in the success rate from 84.7 % after 10 years to 59.1 % after 15 years [16]. The failure events were characterized by complete fracture of the restoration or a veneer chipping or an arrested crack [14–16]. These failures affect the well-being of patients, including both physical and emotional aspects, as well as the financial cost. Improvements in material and design may be recommended if the source of fractures is identified. Crucial information about the fracture event can be gathered with the help of failure analysis tools and techniques that can improve the overall lifetime of these all-ceramic restorations.

**Fig. 1.**
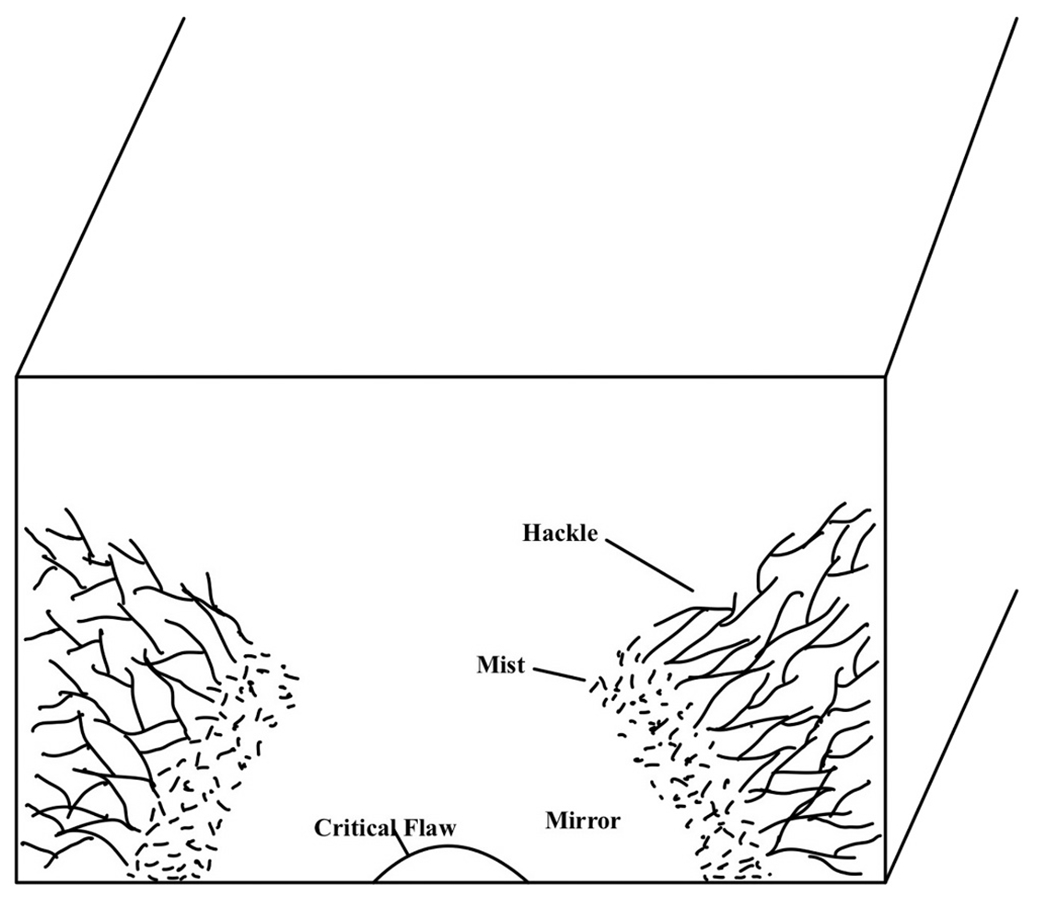
Fracture surface features occurring in brittle materials. The regions are not drawn to scale.

Fracture in brittle materials typically initiates at a pre-existing strength-limiting flaw, such as a microcrack, pore, or inclusion, which acts as a stress concentrator under applied loading. When the local stress intensity factor at the flaw tip exceeds the material’s fracture toughness, the crack propagates rapidly through the specimen, often following the path of least resistance along the microstructural features of the material. In flexural or bending stress configurations, asymmetric loading results in a characteristic crack trajectory: the crack tends to initiate on the tensile side and propagate toward the compressive side. As the crack front approaches the compressive zone, it may decelerate or curve, forming a region called compression curl due to the crack being deflected or arrested by the compressive stresses that oppose further opening [17,18]. This feature is commonly observed in fractographic analyses of brittle materials and provides insight into the stress field and direction of crack propagation at the time of failure. In large or high-strength brittle specimens, the fracture surface often exhibits distinct morphological features that reflect the dynamics of crack initiation and propagation. The fracture process results in the formation of three different regions known as mirror, mist, and hackle [19,20] as shown in Fig. 1. The mirror region is the area immediately surrounding the origin of the fracture and appears relatively smooth and featureless under low magnification. It forms during the early stages of crack propagation when the crack velocity is relatively low and stable, resulting in minimal surface roughness. The size of the mirror region is inversely related to the fracture stress, making it a useful indicator of the applied stress at failure [21,22]. As the crack accelerates and its velocity increases, the fracture transitions into the mist region, which appears slightly roughened or stippled. This region represents a transition zone where small instabilities and micro-branching begin to develop in the crack front, indicating an increase in crack propagation energy. At even higher velocities, the fracture enters the hackle region, which is characterized by a coarse and striated surface morphology. This region forms when the crack front becomes highly unstable, often branching and deflecting due to interactions with microstructural heterogeneities. The hackle marks often radiate outward from the origin and provide insight into the direction of crack propagation. The mist and hackle regions are precursors to macroscopic crack branching [17,23]. Dentistry has long used fractographic techniques to study fractures in ceramic restorations [17].

**Fig. 2.**
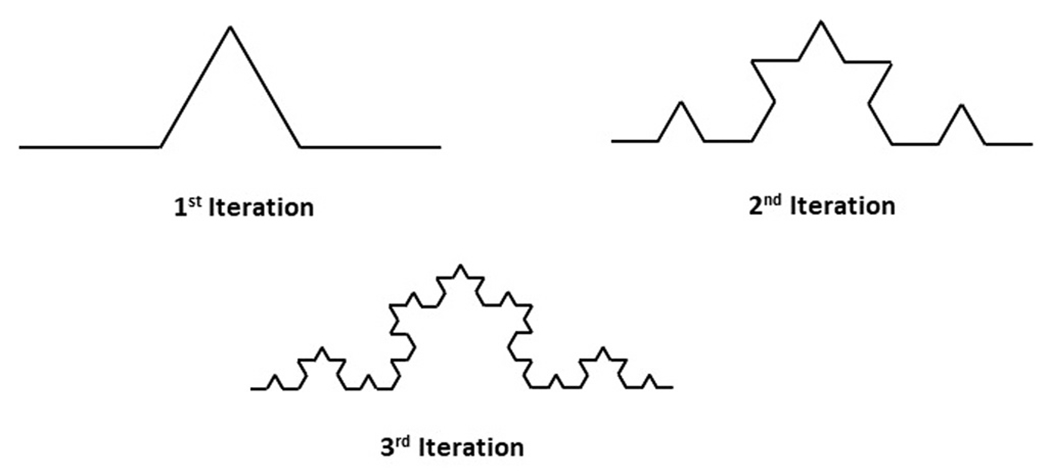
Koch curve representing a fractal curve exhibiting self-similarity. Each segment of the curve replicates the overall shape at a smaller scale.

Qualitative information such as the location of origin of failure and direction of crack propagation can be collected using optical instruments such as loupes and microscopes (optical, laser confocal, and scanning electron) [17]. Whereas, quantitative data can be gathered by calculating the fracture toughness of the material using Griffith and Irwin’s relationship between fracture energy or fracture toughness and critical flaw size and applied stresses [24,25]. Traditional quantitative fractography techniques require the failure origin to assess the factors responsible for the failure. Sometimes, the origin of failure can be lost if the mastication is continued after the fracture, or it can be lost during the retrieval process. The loss of failure origin limits the amount of information about the fracture event that can be collected from clinically failed restorations. However, fractal geometry can be used to estimate fracture toughness even if the failure origin is lost [26–29]. Fractal geometry is a branch of non-Euclidean geometry used to describe complex structures that exhibit self-similarity at multiple length scales, as shown in Fig. 2. Unlike classical Euclidean geometry, which is constrained to integer dimensions (e.g., lines with dimension 1, planes with dimension 2), fractal geometry allows for non-integer dimensions associated with it [30,31]. The fractal dimension can be represented as 2.D*, where 2 represents the Euclidean (flat) surface dimension, and D*, the fractal dimensional increment, represents the amount of fracture surface tortuosity out of plane. The initial relation between fractal geometry of ceramic fracture surfaces and fracture toughness was developed by Mecholsky in a series of studies with several co-workers [30–36]. The relation was later modified by Jodha et al. to account for crack tip shielding mechanisms that occur in some ceramics, such as crack bridging and transformation toughening [26,27]:

(1) $$K_{c}=K_{o}+K_{R}+E\sqrt{a_{o}\;D^{*}}$$

where the mode I fracture toughness (or critical stress intensity factor, K_c_) is the sum of the following contributions: the fracture toughness associated with a flat fracture surface (having D* = 0) is K_0_. K_R_ is the factor accounting for crack tip shielding mechanisms that lead to rising R-curve behavior [26,27]. The final term represents the toughness associated with tortuosity of the surface out of the plane of fracture, and it is composed of Young’s modulus of elasticity (E), the fundamental unit of fracture (or characteristic length, a_0_), and the amount of tortuosity (or fractal dimensional increment, D*). Over the years, researchers have developed various techniques to calculate fractal dimensional increment (D*) [37–41]. Of these techniques, Hill et al. used the Slit-Island Richardson (SIR) method to calculate the fractal dimensional increment of the fracture surface and graph its relationship with the fracture toughness for many ceramics. The researchers observed that ceramics could be classified into three broad categories based on the microstructure: fine-grained polycrystalline ceramics, glasses and glass-ceramics, and single crystal and coarse-grained ceramics [33,34]. The SIR method is labor-intensive and results in biased and imprecise D values as it requires physical sectioning of the fracture surface, and the sectioning plane needs to be parallel to the fracture surface [35]. Key et al. reported the relationship between fracture toughness and the square root of fractal dimensional increment for two benchmark ceramics (silicon nitride and silica glass) that are not used in dentistry. These ceramics are important because they have been thoroughly characterized in terms of fracture behavior [42], which allowed the validation of a method for unbiasing fractal dimension estimates [43]. The technique was later validated for two glass-ceramics that are used in dentistry (IPS e.max CAD and IPS e.max Ceram) as well as a polycrystalline ceramic that is widely used in dentistry (3Y-TZP) [26,27]. As a result of these studies, the fracture toughness of monolithic dental ceramics can now be estimated based on measurement of the fractal dimension of the fracture surface.

An early study showed that the features within the mirror, mist, and hackle regions of fracture surfaces are similar [44]. The optical limitations of microscopes resulted in fractal dimension measurements being performed using a physical sectioning method and limited to only the hackle region [34,35]. The submicron fracture features, such as micro-branching in the mirror and mist regions, could not be measured. However, use of only the hackle region would limit the usefulness of the technique in dentistry because dental implants and restorations have small sizes and often have low failure stress [45–47], which prevents the appearance of a hackle region on the fracture surface. It would be useful to know whether the fractal dimension measured in the mirror and mist regions has the same value as that measured in the hackle region of dental ceramic fracture surfaces. This may be determined using an instrument that can scan the surface in 3D, such as a confocal microscope or an atomic force microscope. Earlier studies to calculate fractal dimensional increment (D*) have reported lower signal-to-noise ratio and higher fractal dimensional increment values for mirror region of the fracture surface.

The objective of this study were:

1. To identify and characterize the mirror, mist, and hackle regions on the fracture surfaces of lithium disilicate glass-ceramic using atomic force microscopy (AFM), hypothesizing that these regions exhibit distinct and reproducible surface morphologies corresponding to different stages of crack propagation – ranging from smooth (mirror) to increasingly complex (mist and hackle).
2. To calculate and compare the fractal dimension values from the mirror, mist, and hackle regions using AFM, based on the hypothesis that the fractal dimension remains consistent across all regions because, as fundamental mechanism of crack propagation are the same, regardless of the macroscopic appearance of the fracture surface, and hence the nanoscale features governing the fractal nature should be the same across the various regions of the fracture surface.

## 2. Materials and methods

Nine rectangular beams of lithium disilicate glass-ceramic (LDS, IPS e.max CAD, Ivoclar Vivadent, Schaan, Liechtenstein) were prepared. A diamond blade (Diamond Cut-off Wheel M0D15, Struers, Cleveland, OH) was used to section blocks with cutting equipment (Accutom-50, Struers, Cleveland, OH) under irrigation. The blade was set to rotate at 300 rpm, and the blocks were fed at 0.45 mm/min. The specimens were crystallized at 850 °C using the firing furnace (Multimat Touch & Press, Dentsply Ceramco, Burlington, NJ) and a crystallization tray with firing paste (IPS Object Fix Flow, Ivoclar Vivadent, Schaan, Liechtenstein) as per the manufacturer’s recommendation. The beams were ground to the final dimensions of 3 × 4 × 32 mm. One of the 3 mm faces was polished using a diamond lapping film (Allied High Tech Products Inc., Rancho Dominguez, CA) with decreasing order of abrasiveness 30 and 15 μm at 250 rpm. A Knoop indenter (Tukon MO, Wilson Instrument, Bridgeport, CT) was used to make an indentation on the polished surface with a load of 10 N. The indentation was made at a lower load than what is recommended in ASTM C1421 [48]. This was done to ensure that all of the fracture regions (mirror, mist, and hackle) would lie within the specimen geometry. The beams were tested in 4-point flexure at a loading rate of 12.6 MPa/s in deionized water until failure. The specimens were tested in water as part of a preliminary study to show no significant effect on the fracture toughness of ceramics when tested in either simulated saliva to mimic the oral environment or water. The failure load (in newtons) was recorded.

An ultrasonic bath (Model 150T, Aqua Sonic, VWR scientific products, Radnor, PA) was used to clean the fractured specimens while submerged in a detergent solution (Patterson Multipurpose Enzyme Tablets, Patterson Dental, Saint Paul, MN). Cleaning was followed by rinsing using deionized water and 190 proof ethanol. One-half of the fracture surface was sputter-coated with 4 nm gold (EM ACE-600, Leica Microsystems, Buffalo Grove, IL). The gold-coated specimens were observed under a scanning electron microscope (SUPRA 40, Carl Zeiss, Thornwood, NY) to identify the mirror-mist and mist-hackle boundaries.

The second half of the fractured surface was used to calculate the fractal dimension in different regions (mirror, mist, and hackle). Two impressions of the fracture surface were taken using a light body poly-vinylsiloxane impression material (PVS, Extrude Base, Kerr Corporation, Orange, CA). As any debris remaining on the surface may have adhered to the first impression, this impression was discarded. A second impression was taken of the entire fracture surface and was allowed to outgas for an hour [26–28,42,49]. A low viscosity, low shrinkage epoxy system (EpoxySet, Allied High Tech Products Inc., Rancho Dominguez, CA) was used to create a positive replica. The epoxy replicas were then carefully placed on polymeric clay (Sculpey III, Polyform Products Company, Elk Grove Village, IL). The epoxy replicas were scanned by an atomic force microscope (AFM, Bioscope Catalyst, Bruker, Santa Bar-bara, CA). Three scans (n = 3 × 9 = 27) were made from each of the mirror, mist, and hackle regions of each fracture surface. The scanned images were leveled using a 1st order flattening operation, followed by noise filtering in Nanoscope Analysis software (Bruker Corporation, Santa Barbara, CA) [26–28,42]. The height sensor data was exported in a.txt format. A custom MathCAD (Parametric Technology Corporation, Needham, MA) script was used to downsize the data to 256 pixels by 256 pixels and convert the.txt file to.sur file. The Minkowski Cover algorithm after bias correction was used to calculate the fractal dimension using the FRACTALS software [26–28,42,43].

A multilevel random intercept with Gaussian family, identity link, and a variance components covariance model was used to compare the fractal dimension values in the mirror, mist, and hackle regions of the fracture surface using Stata (StataCorp, College Station, TX). The significance level was set at α = 0.05.

## 3. Results

**Fig. 3.**
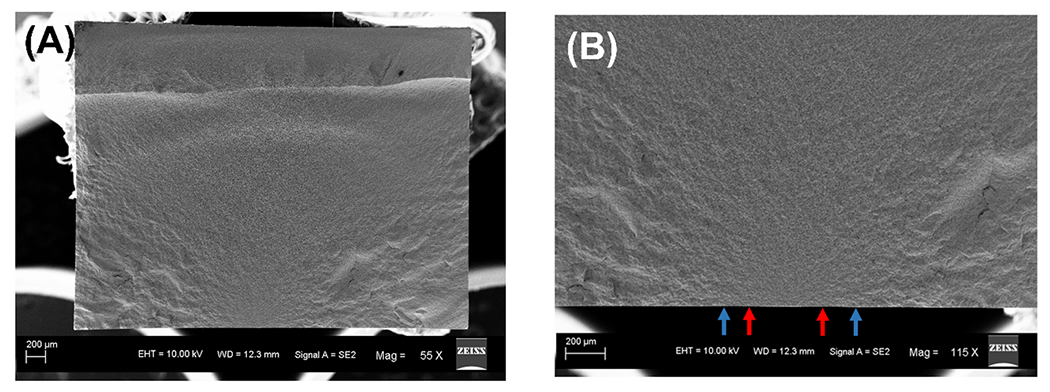
A) Scanning electron micrograph of the fracture surface of LDS specimens, B) 2 × magnification of the image showing mirror-mist boundaries (red arrows), and the mist-hackle boundaries (blue arrows).

**Fig. 4.**
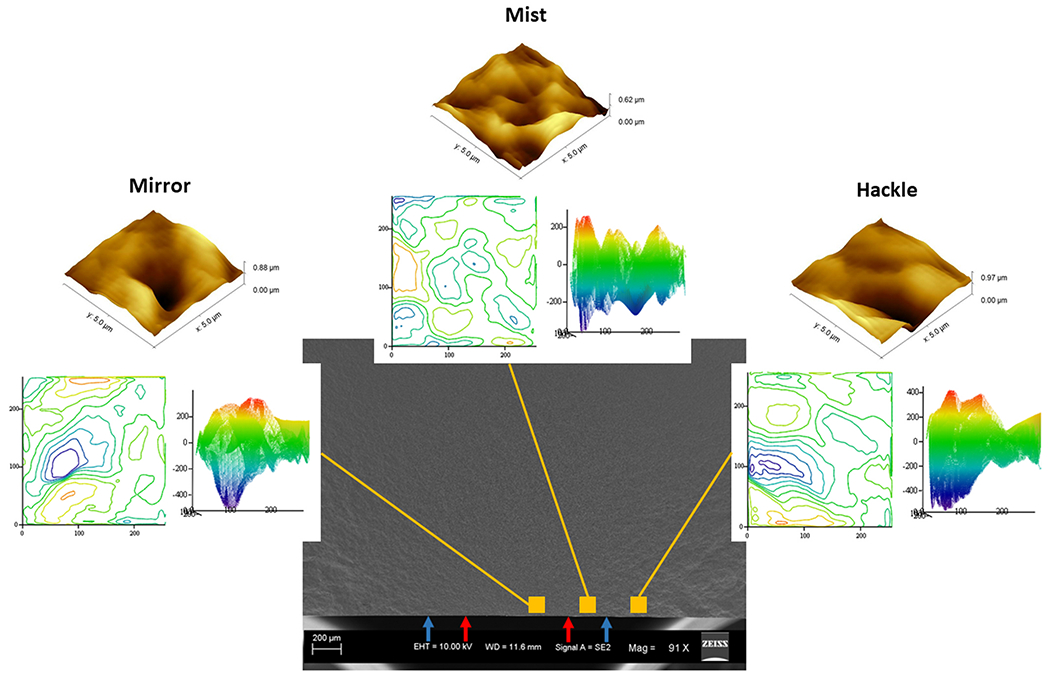
Fracture surface of LDS specimen. The inset shows the contour plot of bird’s eye view and surface projected against the x-z plane for the three regions – mirror, mist, and hackle, and a 3D plot of the AFM scans. The red and blue arrows on the micrographs represent the mirror-mist, and mist-hackle boundaries, respectively. The different colors from blue (low) to red (high) represent the heights of different points on the surface.

**Fig. 5.**
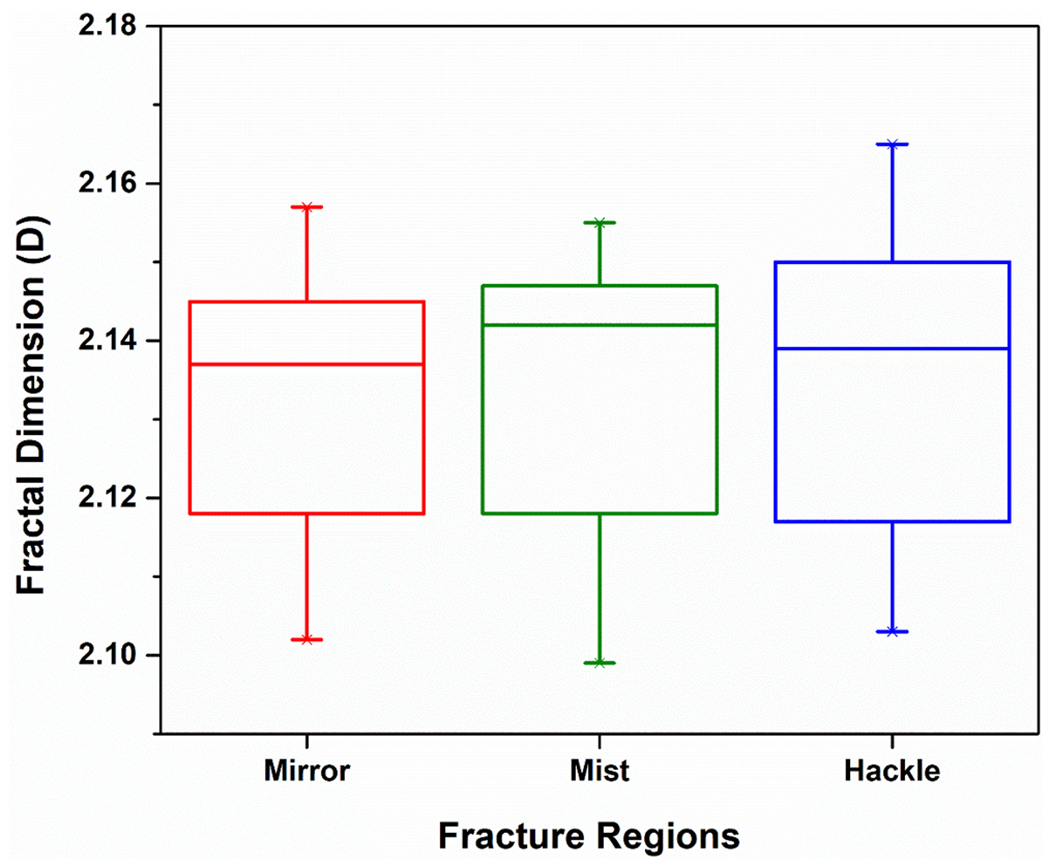
Box plot of fractal dimension from the mirror, mist, and hackle regions of the LDS fracture surface.

The fracture initiated from the controlled flaw on all of the LDS specimens except one, which failed from a surface flaw closer to the edge. The mean failure stress was 201 ± 16 MPa for all specimens. Fig. 3A shows a representative scanning electron micrograph of the LDS fracture surface. Fig. 3B is the magnified version showing the mirror-mist (red arrows) and mist-hackle (blue arrows) boundaries. Fig. 4 shows the different regions of fractal dimension measurement. The median D values (25 %, 75 % quartiles) from the mirror, mist, and hackle regions were 2.14 (2.12, 2.14), 2.14 (2.12, 2.15), and 2.14 (2.12, 2.15), respectively, as shown in Fig. 5. The statistical analysis showed that the fractal dimension between the mirror-mist (p = 0.51), mist-hackle (p = 0.90), and mirror-hackle (p = 0.43) regions are not significantly different.

## 4. Discussion

Fractal analysis can be carried out using various instruments such as optical, confocal, and atomic force microscopes [29]. Optical and confocal microscopes have long z-ranges, but have lower z-resolution and low signal-to-noise ratio. On the other hand, atomic force microscopes can visualize at a nanoscale level but have shorter z-ranges. Jodha et al. observed that the atomic force microscope also has a low signal-to-noise ratio if the surfaces analyzed are smooth [26,42]. Various studies have reported that a noise filtering technique can remove electrical and mechanical noises from AFM scans without affecting the fractal dimension and surface roughness [26,42]. The fractal dimension values in this study are similar to those reported by Jodha et al., D= 2.14 (2.14, 2.15), for the mirror region [26]. Jodha et al. tested lithium disilicate glass-ceramics under four-point flexure at a slower stress rate (9.3 N/s) after introducing controlled flaws at a heavier indentation load (25 N) and calculated the fracture toughness and fractal dimension after noise filtering [26].

A previous study analyzed the different regions of the fracture surface using the transmission electron microscope and observed rounded ridges in the mirror region that elongate gradually in the direction of crack propagation closer to the mirror-mist boundary [44]. Later, another researcher explained that this could be due to the twisting or tilting of the crack front out of the main fracture plane [17]. Beauchamp observed that nano-micro scale hackle lines start to emerge in the mist region that grows into larger tongue-like segments as the crack front deviates further from the fracture plane, leading to micro-steps running parallel to the direction of crack propagation, resulting in a rougher region called hackle [44,50]. The results in the current study that the fractal dimension is the same in the different regions of the fracture surface agree with Beauchamp’s qualitative observations that the features within the mirror, mist, and hackle regions are similar in character but only differ in scale. The hackle are thought to result from the intersections of Wallner lines generated during the dynamic fracture event. These features may arise due to periodic relinking of tilted or twisted segments with the main crack plane. As crack propagates at high speeds, microstructural inhomogeneities or external perturbations can give rise to elastic stress waves that have high velocity and overtake the advancing crack front, and the interaction of these stress waves with the advancing crack front produces Wallner lines – curved markings that record the history of this interaction [17]. Simultaneously, localized instabilities in crack propagation can lead to the development of hackle segments that are misaligned with the main crack path, either through tilting out of the fracture plane or twisting around a perpendicular axis. These misaligned segments concentrate stress along specific regions that may result in the formation of a band of hackle along the crack front [44].

A previous study [51] reported differences in the fractal dimension increment values calculated for the mirror, mist, and hackle regions of fracture surfaces, with the mirror region exhibiting higher values than the other two. Atomic force microscopy (AFM) was used for surface characterization, and the fractal dimension (D) was calculated using two software packages: Gwyddion and WSxM. In Gwyddion, four methods were employed to compute D, but only triangulation and cube counting yielded consistent and reliable results. Among these, the mirror region showed notably large standard deviations in the measured D values.

In the present analysis, the signal-to-noise ratio (SNR) in the mirror region was observed to be lower than in the mist and hackle regions, likely due to the smoother macroscopic topography of the mirror surface. Noise filtering was necessary to eliminate electrical and mechanical interference from the AFM scans. This reduced SNR in the mirror region may account for the elevated D values reported by Smith and Mecholsky [51], as increased noise can artificially inflate fractal dimension values.

However, this study showed that fractal analysis can be performed in any region of the primary fracture surface on lithium disilicate glass-ceramics. The fractal dimension values were not significantly different in the mirror, mist, and hackle regions, which would be particularly useful in clinical failure analysis as any region of the primary fracture surface would provide the same information. The calculated fractal dimension can then be used to estimate the material fracture toughness based on the relationship between the toughness and square-root of fractal dimensional increment [26]. The estimated fracture toughness can be compared with the known toughness for the material. Any deviations in the toughness can point towards the reasons for the failure of the restoration in function. Corrective measures can then be taken to make improvements to the material and design of these restorations to prevent failures in the future.

## 5. Conclusion

The mirror, mist, and hackle regions of the fracture surface were analyzed, and the fractal dimension was calculated. No significant difference was observed between the values obtained from the three different regions in lithia disilicate glass-ceramics, suggesting a consistent scaling behavior across the primary fracture surface. The results here imply that any portion of the primary fracture surface can be analyzed using fractal analysis to investigate the stress state and environmental conditions present at the time of failure. This improves future material strategies as fractal dimension can be used as a robust failure analysis tool to improve the reliability and performance of ceramic in biomedical, aerospace, and structural applications.
